# Gossypium in Dysmenorrhœa

**Published:** 1874-11

**Authors:** 


GOSSYPIUM IN DYSMENORRHŒA.—The following is the formula:

| | |
|---|---|
| ℞.—Fld. ext. Gossypium Herb, . . . | ℥ ij. |
| Fld. ext. Ergot, . . . . . . . . | ℥ ij. |
| Tr. Hellebore Nig., . . . . . . | ℥ ij. |

M. Sig.—Teaspoonful every three hours.

Let the patient commence taking it three or four days before the menses are expected to occur.—*Medical and Surgical Reporter.*

